# Case Report: Anlotinib combined with radiotherapy achieves sustained disease control in advanced malignant melanotic nerve sheath tumor

**DOI:** 10.3389/fonc.2026.1680441

**Published:** 2026-02-03

**Authors:** Huizhan Zhu, Wei Shen, Han Yang, Xiaoyang Han, Weiwei Li, Jinling Xie, Yong Tan, Ping Lu, Yinghua Ji, Yana Zhang

**Affiliations:** 1 Department of Oncology, The First Affiliated Hospital of Xinxiang Medical University, Henan, China; 2 Department of Life Science Research Center, The First Affiliated Hospital of Xinxiang Medical University, Henan, China; 3 Department of Gastrointestinal Surgery, The First Affiliated Hospital of Xinxiang Medical University, Henan, China

**Keywords:** anlotinib, malignant melanotic nerve sheath tumor, metastasis, radiotherapy, targeted therapy

## Abstract

**Background:**

Malignant melanotic nerve sheath tumor (MMNST) is a rare and aggressive subtype of malignant peripheral nerve sheath tumor (MPNST) characterized by melanin-producing cells. Due to its rarity, there is currently no standardized therapeutic strategy, posing significant challenges in selecting effective treatment modalities.

**Case summary:**

We report the first documented case of a 54-year-old female with recurrent MMNST and multiple pulmonary metastases. Following local radiotherapy combined with systemic anlotinib treatment, the patient achieved prolonged progression-free survival (PFS) exceeding 32 months.

**Conclusion:**

This case suggests that anlotinib combined with radiotherapy may be an effective therapeutic option for advanced MMNST, providing valuable clinical evidence for managing this malignancy.

## 1 Introduction

Malignant melanotic nerve sheath tumors (MMNSTs), previously referred to as melanotic schwannomas (MS), are exceptionally rare neoplasms accounting for less than 1% of primary peripheral nerve sheath tumors (1). These tumors exhibit a slightly higher prevalence among females, with a reported female-to-male ratio of 1.4:1 (2). MMNSTs are classified into psammomatous subtypes—characterized by calcified psammoma bodies observed in approximately half of cases—and non-psammomatous subtypes (3). The median age at presentation differs between these subtypes, being approximately 23 years for psammomatous variants and around 40 years for non-psammomatous variants (3). However, no prognostic differences have been identified between these classifications (4). MMNSTs most commonly originate from intracranial structures or posterior spinal nerve roots, but can also arise from less common sites such as the sympathetic chain, auditory nerves, cerebellum, or soft tissues (3). Clinical manifestations vary depending on tumor location and size as well as local compressive effects; pain, weakness, or sensory abnormalities within affected dermatomal regions are frequently reported (5, 6). Diagnosis primarily relies on histopathological examination since standardized treatment protocols remain unavailable.

For localized MMNST, treatment typically involves surgical resection (gross-total resection [GTR] or subtotal resection [STR]) combined with adjuvant radiotherapy (7). STR is a known risk factor for postoperative recurrence and metastasis (8), with reported rates of 35% for local recurrence and 44% for distant metastasis (9); the median time to recurrence or metastasis is approximately 2 years (7). Notably, most MMNSTs present with distant metastases at diagnosis, with the lungs being the most common site (10). Complete resection is often challenging due to high relapse and metastasis rates, and the roles of radiotherapy, chemotherapy, and targeted therapy remain poorly defined (11). Anlotinib is a novel multi-target tyrosine kinase inhibitor that exerts antitumor effects by inhibiting receptors such as *VEGFR1/2/3*, *c-Kit*, *PDGFR-α*, and *FGFR*, thereby disrupting tumor growth and survival signaling pathways (12). It has demonstrated efficacy in various malignancies, including non-small cell lung cancer, small cell lung cancer, soft tissue sarcoma, and medullary thyroid carcinoma (13–18). Here, we report the first case of advanced MMNST with multiple pulmonary metastases treated with anlotinib and radiotherapy, resulting in prolonged disease control.

## 2 Case description

**Figure 1:**
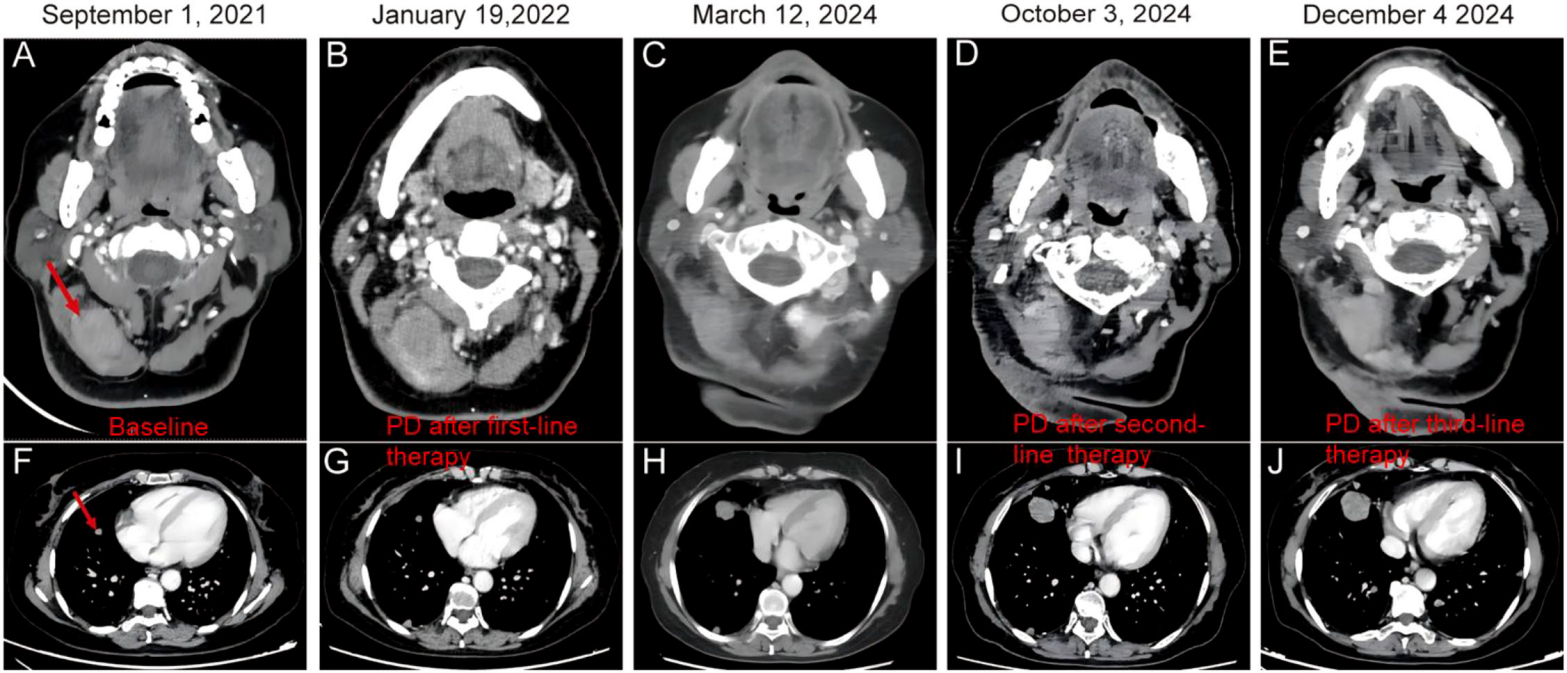
Computed Tomography (CT) images of a 54-year-old female with recurrent malignant melanotic nerve sheath tumor with multiple lung metastases. Serial CT scans of the neck **(A–E)** and chest **(F–J)** demonstrate persistent imaging abnormalities over time. The red arrow showed irregular soft tissue masses in the right posterior neck **(A-E)**. The red arrows show soft tissue density nodules of different sizes in the lungs, which show mild to moderate enhancement in the enhanced phase **(F-J)**.

**Figure 2:**
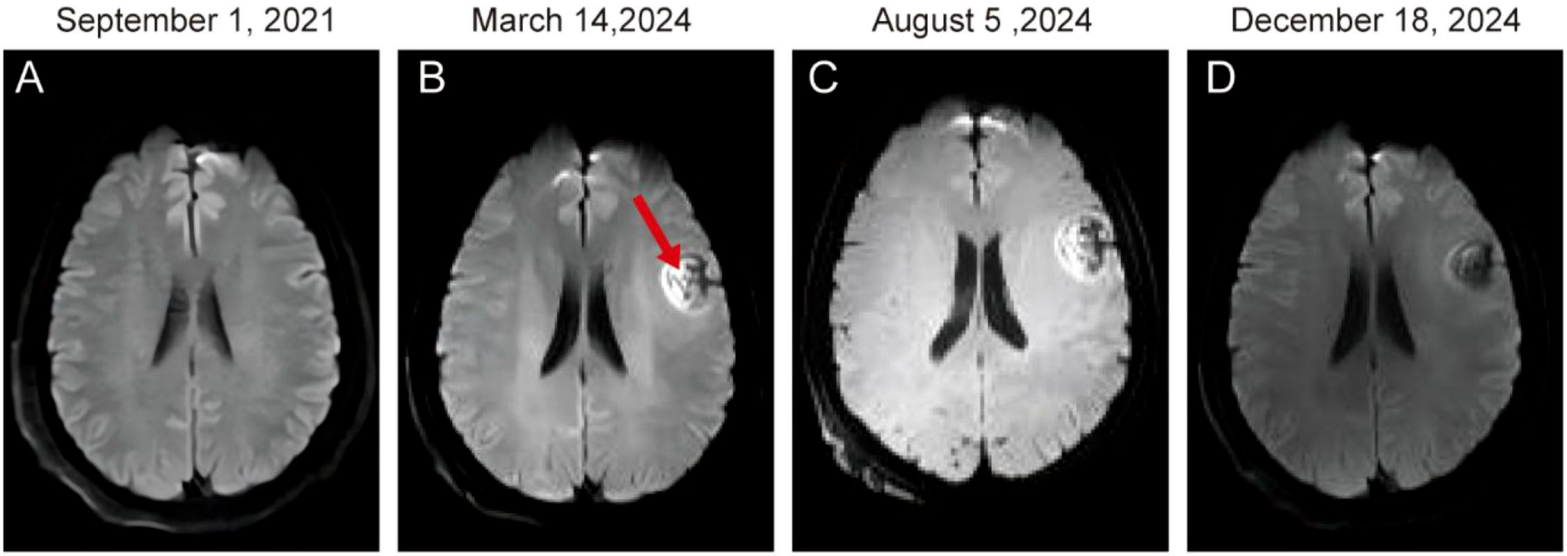
Magnetic resonance imaging (MRI)of a 54-year-old female with recurrent malignant melanotic nerve sheath tumor with brain metastases. Diffusion-weighted image (DWI) shows mixed signal intensity of the right frontal **(A-D)**.

A 54-year-old female presented with a right neck mass without other associated symptoms. She reported no recent illness or changes in medications, diet, or daily routine. On July 28, 2021, ultrasonography revealed enlarged cervical lymph nodes. A lymph node biopsy performed on August 17, 2021, confirmed MMNST. Histologically, hematoxylin-eosin (HE) staining showed spindle and epithelioid tumor cells with prominent melanin. Immunohistochemistry (IHC) demonstrated positivity for *S100*, negativity for *Melan-A* and *HMB45*, and a *Ki-67* index of 0%. A neck and chest computed tomography (CT) scan on September 1, 2021, identified a 22.8 × 26.8 × 32.7 mm neck mass and multiple bilateral pulmonary metastases (**Figures 1A, F**). Brain magnetic resonance imaging (MRI) showed no intracranial metastases (**Figure 2A**).

The patient began first-line treatment with four cycles of doxorubicin, ifosfamide, and bevacizumab, completed on November 11, 2021. On December 7, 2021, she developed Grade IV myelosuppression, prompting a regimen adjustment to “doxorubicin + bevacizumab” until December 29, 2021, with no other significant adverse events.

Follow-up imaging in January 19, 2022 showed disease progression, with the neck mass enlarging to 39.0 × 26.0 mm (**Figures 1B, G**). The patient initiated anlotinib (12 mg/day for 14 consecutive days in a 21-day cycle) combined with hyperfractionated radiotherapy to the neck mass (total dose: 52.5 Gy in 15 fractions), completed by March 7, 2022. On March 14, 2024, the patient was readmitted with worsening dizziness and nausea. CT scans showed stable neck and lung lesions (**Figures 1C, H**), but brain MRI revealed a new left frontal lobe metastatic lesion (25.8 × 20.5 × 23.7 mm; **Figure 2B**), which was treated with stereotactic radiotherapy (SRT; 48 Gy in 12 fractions). Anlotinib maintenance therapy continued.

**Figure 3:**
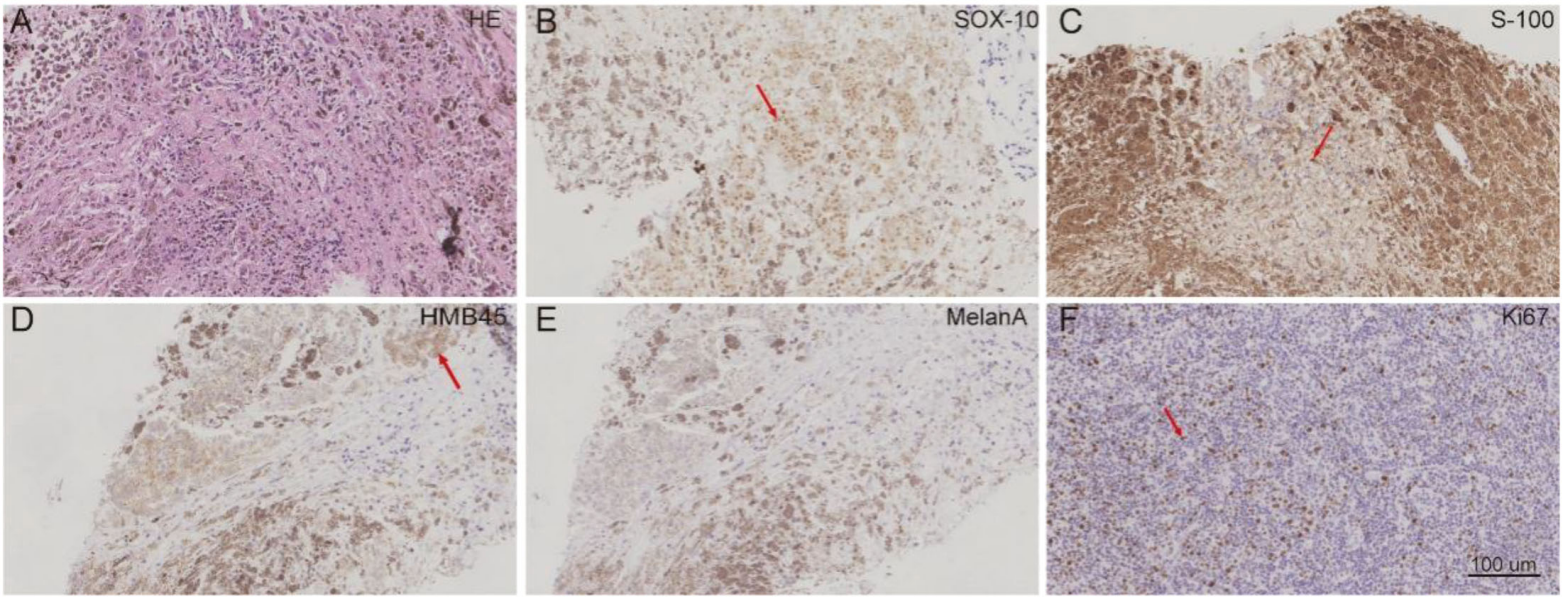
Histopathological and immunohistochemical findings of pulmonary metastases in a 54-year-old female with recurrent MMNST. Microscopic examination revealed a proliferation of spindle-shaped and epithelioid tumor cells containing abundant cytoplasmic melanin **(A)**, arrows). Immunohistochemical staining demonstrated diffuse positivity for *SOX10* and *S100* protein **(B, C)**, focal positivity for *HMB45***(D)**, arrow), and negativity for *Melan-A***(E)**. The *Ki-67* proliferation index was approximately 2% **(F)**, arrow).

**Figure 4:**
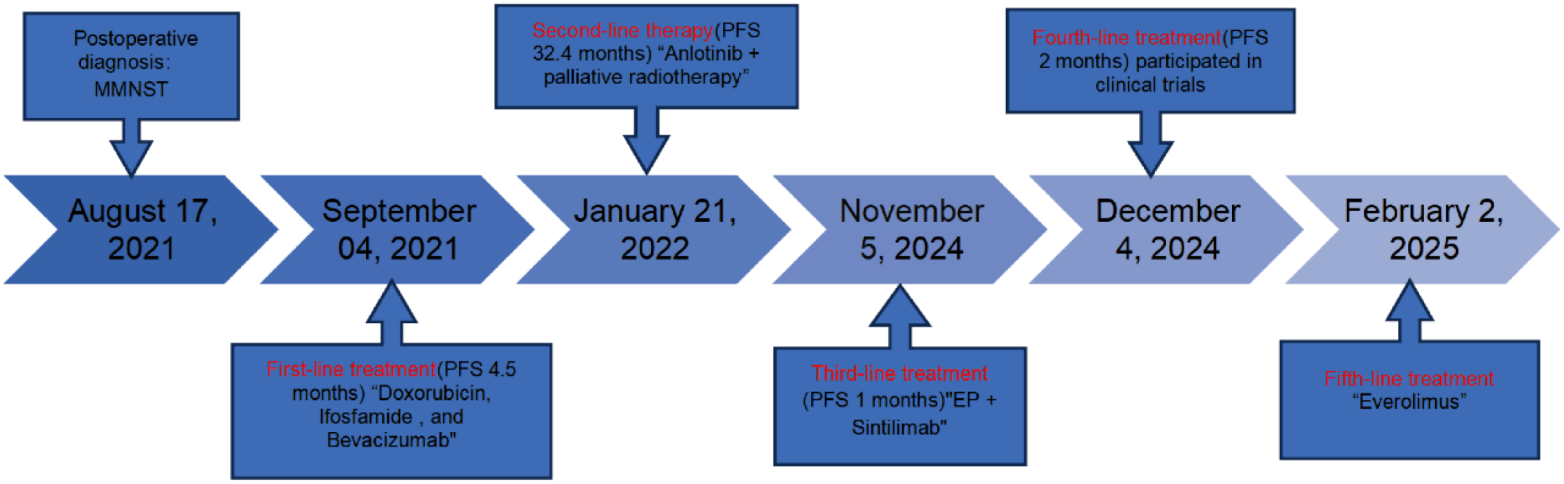
Overview of the whole treatment process.

On October 3, 2024, imaging detected a new liver metastasis and progressive enlargement of lung lesions (**Figures 1D, I**). A lung node biopsy on November 1, 2024, confirmed MMNST metastasis with IHC positivity for S100 and SOX-10, patchy positivity for *HMB45*, negativity for *Melan-A* and *TTF-1*, and a *Ki-67* index of 2% (**Figures 3A-F**). The patient’s follow-up examination shows stable lesions at the head metastatic site (**Figure 2C**). The patient initiated with cisplatin, etoposide and sintilimab on November 5, 2024, but December 4, 2024, imaging revealed further progression of lung lesions (**Figures 1E, J**, **2D**). She subsequently enrolled in a clinical trial for targeted therapy.​On February 2, 2025, CT scans identified multiple liver metastases, and everolimus was initiated (**Figure 4**).

## 3 Discussion

MMNST is associated with a poor prognosis due to its high rates of recurrence and metastasis (1, 19). Previous studies have speculated that it may be related to genetic factors, ionizing radiation, or malignant transformation of neurofibromas (6). Diagnosis is challenging due to histological overlap with melanocytic tumors, both of which exhibit spindle/epithelioid morphology, pigmentation, and *S100* expression (11). Key distinguishing features include MMNST’s Schwann cell origin, well-defined borders, rare nuclear pleomorphism, and frequent positive for *HMB45, Melan-A, S100, SOX10* and *Vimentin* (20). Additional discriminants include *Ki-67* index, lobular/clustered tumor cell arrangement, syncytial features, psammoma bodies, and fat accumulation. Radiologically, MMNSTs typically show T1 hyperintensity and T2 hypointensity on MRI due to intratumoral melanin (11), aiding in the differentiation from melanoma. MMNST is sometimes considered an intermediate entity between schwannomas and malignant melanoma (21), but in our case, histological consistency with MMNST was maintained throughout progression, with no evidence of melanoma transformation. Prognosis remains dismal, with a typical survival of less than 1 year (22), highlighting the need for effective therapies.

For metastatic MMNST, ifosfamide plus doxorubicin is the most reported chemotherapy regimen, while fotemustine has shown partial remission in one case (23). However, chemotherapy response rates are low, with limited impact on mortality (5). In our patient, first-line doxorubicin/ifosfamide/bevacizumab failed to control disease, possibly due to Grade IV myelosuppression requiring dose reduction.

Anlotinib’s efficacy in MPNSTs was recently suggested by Chen (24), who reported favorable safety and short-term efficacy with denosumab, chemotherapy, and sequential anlotinib. To our knowledge, this is the first report of anlotinib monotherapy (with radiotherapy) in MMNST, achieving a PFS of more than 32 months–markedly longer than the typical survival for advanced disease. Notably, no Grade ≥3 adverse events were observed, consistent with anlotinib’s known safety profile (e.g., Grade 1–2 triglyceride elevation, hypertension, hand-foot syndrome, oral mucositis, and fatigue) (12).

The prolonged disease control observed in this patient is likely attributable to the synergistic interplay among anlotinib’s multifaceted antitumor mechanisms. On one hand, its therapeutic efficacy arises from direct inhibition of angiogenesis mediated by *VEGFR/PDGFR* and tumor cell proliferation driven by *c-Kit/FGFR* pathways that are potentially active in MMNSTs. Notably, anlotinib also has been shown to suppress the extracellular regulated kinase (*ERK*) signaling pathway and reverse the epithelial-mesenchymal transition (EMT) process in various tumor models (25). Additionally, as a molecular characteristic gene of some malignant melanotic nerve sheath tumors (MMNSTs), *PRKAR1A* can drive the *ERK* signaling axis in other tumors (26). On the other hand, anlotinib may also exert indirect effects by promoting vascular normalization and remodeling the tumor immune microenvironment. Recent studies have revealed that anlotinib downregulates transferrin receptor (*TFRC*) expression via the *VEGFR2/AKT/HIF-1α* signaling axis, thereby enhancing infiltration of immune cells such as *CD8^+^* T lymphocytes into hepatocellular carcinoma tissue, ameliorating hypoxia, and reversing immunosuppression (27). In esophageal cancer, anlotinib has also been reported to potentiate the antiangiogenic effects of radiotherapy through inhibition of the *EphA2* pathway (28). Although the relevance of these mechanisms in MMNST remains to be fully elucidated, we hypothesize that, in this case, anlotinib contributed to durable disease control not only through its established antiangiogenic and antiproliferative actions but also by promoting vascular normalization, reshaping the immune microenvironment, and potentially modulating the *PRKAR1A/ERK* axis. Together, these interconnected mechanisms may constitute the molecular basis for the patient’s long-term clinical response.

This study has inherent limitations. As a single case report, it is constrained by a limited sample size and lacks systematic biomarker profiling. Consequently, we are unable to mechanistically validate the proposed signaling pathways or fully explain the patient’s unique PFS pattern and potential heterogeneity in treatment response.

## 4 Conclusion

This case demonstrates that anlotinib, combined with radiotherapy, can achieve prolonged PFS (>32 months) in advanced MMNST with multiple metastases, with a favorable safety profile. These findings offer a new therapeutic strategy for this rare, aggressive malignancy and warrant further investigation in larger cohorts.

## 5 Patient perspective

Written informed consent was obtained from the patient for publication of clinical details and imaging.

## Data Availability

The raw data supporting the conclusions of this article will be made available by the authors, without undue reservation.
